# Touch uses frictional cues to discriminate flat materials

**DOI:** 10.1038/srep25553

**Published:** 2016-05-06

**Authors:** David Gueorguiev, Séréna Bochereau, André Mouraux, Vincent Hayward, Jean-Louis Thonnard

**Affiliations:** 1 Institute of Neuroscience, Université catholique de Louvain, B-1200, Brussels, Belgium; 2 Sorbonne Universités, UPMC Univ Paris 06, UMR 7222, ISIR, F-75005, Paris, France; 3 Cliniques Universitaires Saint-Luc, Physical and Rehabilitation Medicine Department, Université catholique de Louvain, B-1200, Brussels, Belgium

## Abstract

In a forced-choice task, we asked human participants to discriminate by touch alone glass plates from transparent polymethyl methacrylate (PMMA) plastic plates. While the surfaces were flat and did not exhibit geometric features beyond a few tens of nanometres, the materials differed by their molecular structures. They produced similar coefficients of friction and thermal effects were controlled. Most participants performed well above chance and participants with dry fingers discriminated the materials especially well. Current models of tactile surface perception appeal to surface topography and cannot explain our results. A correlation analysis between detailed measurements of the interfacial forces and discrimination performance suggested that the perceptual task depended on the transitory contact phase leading to full slip. This result demonstrates that differences in interfacial mechanics between the finger and a material can be sensed by touch and that the evanescent mechanics that take place before the onset of steady slip have perceptual value.

Tactile sensing plays a key role in our interaction with the world around us. We constantly need to assess the nature of the objects we are in contact with in order to go about our daily lives. Thus far, it has been shown that many manipulative and perceptual tasks are performed on the basis of gross shape^1^,^2^ of frictional properties^3^ and of surface topography^4^,^5^. Tactile mechanics provide cues about the physical properties of touched objects, including their shape^6^, their softness^7^ or their friction^8^. Therefore, objects made of different materials but having similar shapes, frictional properties, and surface topography represent a challenge for the sense of touch. Under these conditions, objects are generally assumed to be discriminated on the basis of their mass properties or thermal properties^9^,^10^. To our knowledge, however, it was never suggested that differences in the materials we touch could be detected on the basis of molecular-level properties.

Water is almost universally present in our environment. With rare exceptions, all surrounding objects are naturally coated with water and water represents approximately 60% of the mass of our bodies. Water has specific molecular-level bonding properties, which manifest themselves by the hydrophobic or hydrophilic character of materials. For example, the dew on grass leaves owes its existence to the hydrophobicity of the leaves. The skin’s *stratum corneum*, which covers our fingers, is mainly hydrophobic but also exhibits permeation properties^11^ and moisture endows it with peculiar frictional properties that influence its contact mechanics with surfaces^12^.

Most previously studied surface-related tactile perceptual tasks rely on sliding fingers on surfaces because discrimination performance is dramatically improved during dynamic exploration^13^. The primary reason for this enhanced performance is that the mechanical fluctuations induced by sliding bring rich information about the underlying topography of a surface compared to the minute skin deformations induced by static contacts. As shown later, in the limit case of perfectly flat surfaces, complex mechanical fluctuations still take place for a large range of loads and sliding velocities. These fluctuations take place during sliding as well as during the transitory phases between sticking and slipping.

Recent studies have shown that the transition from static to dynamic finger-glass contacts can lead to surprisingly complicated dynamics where contacts can fail progressively or catastrophically under load^14^,^15^,^16^ owing to the nonlinear nature of friction and skin biomechanics. Here, we hypothesized that this dynamics could enable humans to discriminate between surfaces that are identical in all their physical aspects, except their molecular structure.

We tested this hypothesis in two experiments where we asked human participants to discriminate between flat surfaces made of glass or made of polymethyl methacrylate (PMMA) (see Supplementary Fig. 1). The samples were thermally equalised through bonding to aluminium blocks acting as heat sinks. We established in experiment 1 that discrimination between the two materials was possible. In experiment 2, we reproduced the task and recorded the contact forces with exacting accuracy together with the psychophysical performance of the participants. The two experiments differed also in the thermal conditions. In experiment 1, the temperature of the samples was kept constant at a value near the natural fingertip temperature. In experiment 2, the samples were at room temperature.

## Results

### Experiment 1

**Figure 1:**
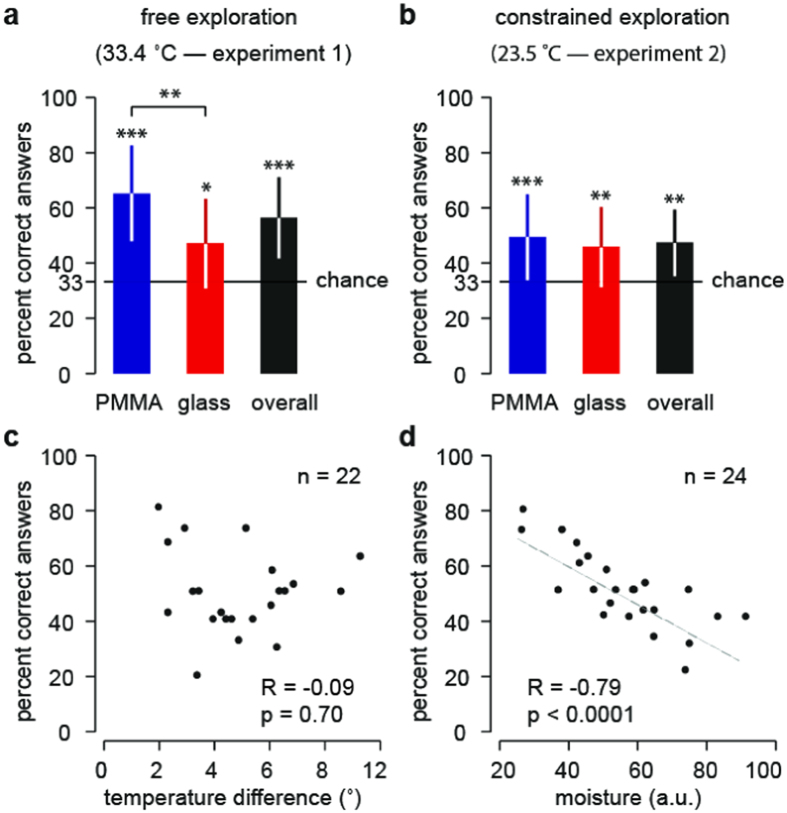
Psychophysical measurements. (**a**) Performance at the task presented as Mean ± s.d. for experiment 1. (**b**) Performance at the task presented as Mean ± s.d. in experiment 2. (**c**) The mean difference of temperature between the fingertip and the samples plotted against performance for all participants of both experiments. (**d**) Mean moisture level of the fingertip plotted against individual performance for all participants of both experiments.

Three samples were placed in a single row in front of the participants. Of the three samples, one was always different from the other two. Participants could explore the samples freely as many times as they wanted before reporting which was different from the other two. The samples were kept at a constant temperature similar to the average temperature of the skin (33.4 ± 0.9 °C; mean ± s.d.). Performance at the task was significantly better than chance level (one sample t-test: t(11) = 5.41, p = 0.0002, 95% CI [13.66, 32.38 ]). (Fig. 1a). The results also suggested that participants were better at identifying the PMMA plate among two glass plates as compared to identifying the glass plate among two PMMA plates. We could rule out this bias to be the result of unseen imperfections in their surface because the answers were consistent across the plates and showed no bias towards a specific plate (Supplementary Fig. 2).

### Experiment 2

The samples were placed in one single column in front of the participant. They were kept at room temperature (23.5 ± 1.5 °C; mean ± s.d.). The participant’s hand was guided by the experimenter to the starting point of each exploration to ensure that the samples were explored from the most distal to the most proximal. The participants’ movements were constrained in order to allow the collection of mechanical data. Each individual sample was explored from left to right in a single swipe. Participants could repeat the exploration up to five times per trial before reporting a judgment. Despite these constraints, performance was also highly significant (one sample t-test: t(11) = 4.16, p = 0.0016, 95% CI [6.836, 22.17]). (Fig. 1b) and there was no significant drop of performance difference between Experiment 1 and 2 (unpaired t-test: t(22) = 1.549, p = 0.136, 95% CI [−19.92, 2.883 ]). The possibility that detection was mediated by an imperfection of plate surface was also assessed (Supplementary Fig. 3) but the answers showed no bias towards a specific plate.

We specifically tested for the possibility that discrimination relied on thermal cues. To this end, in the two experiments and for each participant we recorded the mean temperature difference between the fingertip and the sample. If there was a substantial possibility for thermal cueing, discrimination performance could be expected to be dependent on the amount of thermal difference between the fingertip and the plates. The Pearson’s correlation coefficient between the temperature differences and the individual performances showed non-significant trends corresponding to a slight anti-correlation in experiment 1 (n = 12, R = −0.30, p = 0.33) in which the samples were warmer and a slight correlation in experiment 2 (n = 10, R = 0.62, p = 0.06) in which the samples were colder. Since the distribution of temperature differences was similar in the two experiments (unpaired t-test: t(20) = 1.386, p = 0.18, 95% CI[−0.6722, 3.336]), we computed the overall Pearson’s correlation coefficient between the temperature differences and the individual performances, and no correlation was found (n = 22, R = −0.09 and p = 0.70) (Fig. 1c). These opposite trends for the two experiments rather suggested an effect of the absolute temperature of the fingertip and indeed, higher finger temperature significantly correlated with a better capacity to discriminate the materials (Pearson’ correlation for n = 22, R = 0.47 and p = 0.026). This result was in accordance with previous studies indicating lower mechanical detection thresholds when the fingers are warm^17^,^18^. These results are therefore consistent with a discrimination strategy based on mechanical cues, rather than with a strategy based on thermal cues.

The moisture level of the fingertips was also monitored. We found the moisture level to be strongly anti-correlated with individual performance in experiment 1 (Pearson’s correlation for n = 12, R = −0.90, p < 0.0001) as well as in experiment 2 (Pearson’s correlation for n = 12, R = −0.63, p = 0.028). Since the distributions were similar (unpaired t-test: t(22) = 0.1875, p = 0.85, 95% CI[−12.75, 15.29]), we tested the correlation between moisture level and performance across the two experiments. The correlation was robust (Pearson’s correlation for n = 24, R = −0.79, p < 0.0001) (Fig. 1d). The strong influence of fingertip moisture on performance discrimination motivated us to investigate further the connection between the perceptual performance and the underlying contact mechanics.

**Figure 2:**
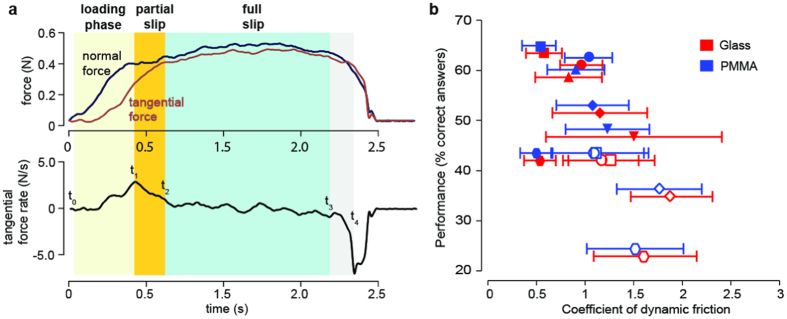
Trial segmentation and coefficient of dynamic friction. (**a**) The rate of change of the tangential force component enabled the extraction of the objective markers t_1_, t_2_, t_3_ and t_4_ (see Materials and Methods), which were used to define the different phases within a swipe: loading phase (t_0_-t_1_), partial slip (t_1_-t_2_) and full slip (t_2_-t_3_). (**b**) The coefficient of dynamic friction, Mean ± s.d., was not distinguishable between materials. The 10 pairs of symbols represent the glass (red) and PMMA (blue) values for each participant. Although the overall individual performance for both materials is identical for the two datasets, the data for glass are slightly shifted downwards for better visibility.

We first verified that, during non-constrained exploration, the behavioural parameters (speed of exploration, normal force applied) did not correlate with individual performance (see Supplementary Fig. 4). We then segmented each swipe into three epochs: a loading phase during which the finger contact is stuck, a partial slip phase during which the outer part of the contact area starts slipping^14^ and a full slip phase during which the contact area is fully sliding (Fig. 2a and materials and methods). The aim of this segmentation was to determine which epoch provided the greatest differentiation between the materials being touched.

**Figure 3:**
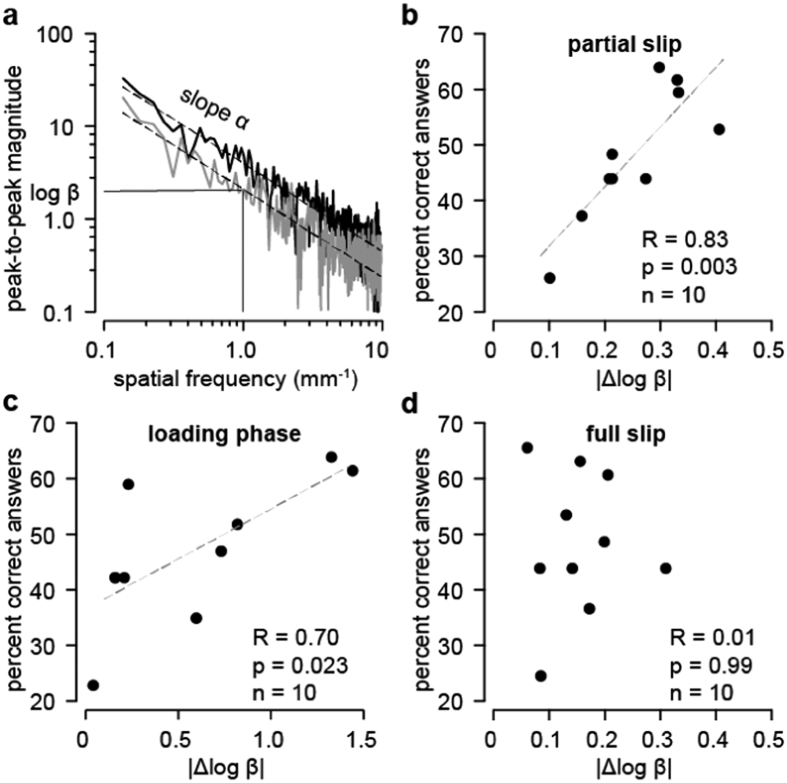
Analysis of the frictional fluctuations. (**a**) Comparison of the amplitude spectra of the tangential force component in the spatial domain. Linear regression gave parameters α and logβ. The differences Δα and Δlogβ between the mean spectra of the two materials were computed for all participants. (**b–d**) The differences in the frictional noise |Δlogβ| between glass and PMMA plotted for each phase against performance: (**b**) Partial slip. (**c**) Loading phase. (**d**) Full slip.

We computed the dynamic coefficient of friction for the two materials defined as the ratio of the magnitude of tangential and normal forces generated by the interaction during full slip for all trials (Fig. 2b). The values of the coefficient of friction varied across participants with a mean value of 1.13 ± 0.13 (mean ± s.d.) but a Mann-Whitney test with a Bonferroni correction did not show significant intra-individual differences related to the materials: nine participants had p-values > 0.05 and one participant, whose performance was average (42,9% of correct answers) had a p-value = 0.042. The coefficients of friction between the two materials were significantly anti-correlated with individual performance (Pearson’s correlation for n = 10, R = −0.67, p = 0.036): discrimination performance was, on average, better when the difference in coefficient of friction was small. This indicates that discrimination did not rely on differences in the average dynamic coefficient of friction during full slip, but does suggest a possible role of frictional dynamics during other phases of the exploration. We thus analysed the spectral characteristics of the forces during the different phases of the sliding. For this purpose, the signals were analysed in the spatial domain such as to characterize the fine fluctuations of force independently of the net slip velocity^19^,^20^. As typically is the case, the spatial frequency amplitude spectrum followed a power law relationship^21^, *T* = βη^α^, where β is a magnitude factor and α quantifies the decay of amplitude with frequency (Fig. 3a).

The spectra were averaged within participants for all trials and the magnitudes and decays were estimated. For each of the three epochs, the spectrum in the range 10^−1^−10 mm^−1^ was compared between glass and PMMA. There was no significant difference in spectral decay between the materials but the magnitudes differed (Δlogβ, Fig. 3b–d). Discrimination performance was strongly correlated with |Δlogβ| during partial slip (Pearson’s correlation for n = 10, R = 0.83, p = 0.003) and moderately during the loading phase (Pearson’s correlation for n = 10, R = 0.70, p = 0.023). On the other hand, correlation was very weak during fully developed slip (Pearson’s correlation for n = 10, R = 0.007, p = 0.99). These correlations between the transient frictional dynamics observed during the early stages of a swipe and individual performance suggest that, during these stages, frictional dynamics of the interactions between the fingertip and the two materials differed, and that this difference enabled the participants to discriminate the two materials.

## Discussion

Performance within our sample of participants was significantly above the chance level in the two experiments, showing that tactile discrimination between two flat materials is possible even in the absence of thermal and surface topography differences. If flat surfaces could be distinguished on the sole basis of frictional cues, then differences must have arisen from molecular-scale properties influencing the interfacial contact of the skin-material pair, in turn influencing its frictional dynamics. The moisture content of the fingers strongly correlated with performance across the study, suggesting that the interaction of water either with the skin tissue or with the skin-material interface played an important role in the tactile discrimination of these two flat surfaces.

During steady sliding, there was no measurable difference in contact mechanics (coefficient of friction, spectral properties of force fluctuations) between the two materials. In contrast, there were significant differences in the magnitude of the interfacial force variations during the partial slip phase corresponding to a progressively failing contact. The partial slip phase comprises a mix of adhesion and slip where, typically, an annulus of failure grows from the periphery of the contact region to eventually invade the whole contact^14^ (see also Supplementary Movie 1). The present measurements corroborated the earlier observation that partial slip tends to last from 100 ms to 300 ms in finger-glass contacts^15^. During this period, the contact evolution can sometimes take the form of a catastrophic failure once a critical load is reached. This finding suggests that the evolution of the contact state, owing to the nonlinearity of friction, can be critically sensitive to small changes in the frictional behaviour of the skin-surface interface.

The observed macroscopic effects must take their roots in smaller-scale physics. The moisture level of the fingertip was already shown to dramatically affect the time course of contact failure under load and thus could explain the performance dependency that we observed between fingertip moisture and discrimination performance^14^. The surfaces that we employed in the present study differed by their hydrophobicity. Ordinary uncoated glass has a hydrophilic, amorphous solid atomic structure and PMMA is a hydrophobic transparent polymer. The other material of the contact pair, the skin *stratum corneum*, is mostly made of keratin, a natural glassy polymer with strong affinity with water. As reviewed recently^22^,^23^, water plasticizes the *stratum corneum* and reduces its elasticity leading to combined adhesion mechanisms. A first mechanism is associated with capillary forces at small normal loads and short time scales^24^, a second is due to plasticization for longer time scales^25^, and others to molecular interactions. The difference in material hydrophobicity is likely to affect these phenomena differentially and their relative contributions could impact the evolution of the stick-to-slip dynamics. At onset, the finger would tend to stick more on glass than on plastic owing to a stronger influence of capillary forces in the early stages of a tangentially loaded contact. Subsequently, during the partial slip phase, higher magnitude of frictional dynamics for PMMA could arise from the rate of breaking of the molecular bonds during the progressive failure of adhesion, which is akin to crack formation. Plasticization occurs over a longer time course and it is unlikely to play a direct role during the early transient phase of tactile exploration but the free exploration allowed in experiment 1 also took place on a longer time course than single swipes. Therefore, different plasticization dynamics might be responsible for the difference in discrimination performance between the two materials, which we observed in experiment 1. The strong effect of hydration of the fingertip could also influence the transmission of subtle frictional fluctuations through variations in skin damping.

The onset of slip received no attention until now since it was hitherto assumed that the tactile perception of materials relied predominantly on interactions taking place over extended periods of full slip. Classical studies of touch focus almost exclusively on rigid surface features without consideration of chemistry of the materials^26^. Our results suggest that other properties affecting frictional dynamics must be taken into consideration, and justifies the current shift toward studies involving naturalistic surfaces^27^,^28^. Moreover, recent reports of astonishing tactile discrimination performance with textured surfaces at the nanometre scale^29^ could be explained, not by direct differences in topography among these surfaces, but rather by the impact that small scale features have on interfacial frictional dynamics.

## Materials and Methods

The ethics committee on human research of Université catholique de Louvain approved the study (Virtual Prototyping of Tactile Displays, PROTOTOUCH-317100). All participants gave written informed consent. The investigation conformed to the principles of the Declaration of Helsinki and experiments were performed in accordance with relevant guidelines and regulations.

### Participants

Data were collected from 24 healthy volunteers (12 in experiment 1 and 12 in experiment 2) aged between 22 and 61. Participants were blindfolded and white noise was played at a comfortable listening level through headsets in order to mask auditory cues.

### Materials

The samples were made of ordinary glass (clear glass, Leroy Merlin, France) or of polymethyl methacrylate (PMMA) plates (Plexiglas^®^, ABP Beaumont plastique, France). Surfaces were perfectly flat. They were chosen for their differences in atomic structure and hydrophobicity. The topographical profiles were assessed with a digital holographic microscope (Lyncée Tec, Switzerland) to check the flatness and absence of topographic features (see Supplementary Fig. 1). The samples dimensions were 60 × 30 × 2 mm. According to findings from previous studies, the differences in the thermal properties of PMMA (heat capacity: 1460 j/kg, thermal conductivity: 0.2 j/m, Contact Coefficient: 0.6 10^3^ J/m^2^s^1/2^K) and glass (heat capacity: 837 j/kg, thermal conductivity: 0.78 j/m, Contact Coefficient: 1.3 10^3^ J/m^2^s^1/2^K) were insufficient to provide thermal cues for the discrimination of the samples or noticeable thermal changes to the fingertip^9^,^30^,^31^. Furthermore, the temperatures of the samples were kept equalised by bonding all triplets of samples to 10 mm thick aluminium blocks acting as heat sinks. The edges were protected with adhesive tape to prevent the participants from using them as a source of information. In experiment 1, the samples were warmed using an infrared heat lamp and thermocouples. The thermocouples were located under the plates (OS36, OMEGA engineering Inc.) and controlled by a thermo-controller (MICROMEGA CN77000, OMEGA engineering Inc.) in order to keep the temperature of the samples constant throughout the whole experiment and match the average temperature of the human fingertip. This procedure could not be implemented in experiment 2, because the setup for temperature control could not be combined with the force sensing plates used for the fine measurements of force dynamics.

### General procedure

The procedure was a forced choice task with three alternatives (3-AFC) where one sample differed from the other two (“odd-man-out”). This procedure was chosen because we did not have prior assumption of the relevant tactile information that could be used to discriminate the surfaces, and it encouraged the participants to search for any subtle differences. The participants washed their hands with commercial liquid soap and waited five minutes before starting the task in order to stabilize the frictional properties of the skin. Three randomly placed samples were presented to the participant (either two samples of PMMA and one sample of glass, or one sample of PMMA and two samples of glass). The participants explored the samples with the index finger of their dominant hand and reported which of the three samples differed from the others two. At the beginning of the experiment, the samples were cleaned with commercial cleaner for glasses (Si Clair, SI International, France) and checked for imperfections. They were additionally quickly cleaned with microfiber cloth every three trials. The performance of each participant was given by the percentage of correct answers and participants were asked about possible imperfections on the samples. An experiment comprised three blocks of fourteen trials. Before and after each block, the temperature of the samples and of the fingertip was assessed with an infrared thermometer (Raytek MI3-M, Raytek, USA). In experiment 2, the temperature values could not be collected for two participants because of a temporary unavailability of the recording device. The moisture level of the fingertip was also measured by averaging three repeated measures using a corneometer (CM 825, Courage + Khazaka electronic GmbH, Germany).

### Force measurements

In experiment 2, the contact forces between the finger and the samples were recorded by a sensitive force sensing plate designed in-house on which the samples were clamped. The force plate measured the normal force components using two load cells (9313AA1, Kistler AG, Switzerland) and the tangential force component using one load cell (9217A, Kistler AG, Switzerland). The offset of the force plate was 1.0 mN for the tangential component of the force and 40 mN for the normal component. The signals were digitized at 10 kHz. The location of the fingertip contact on the plate was estimated by computing the position of the centre of pressure. The force transducer temporarily lost calibration during the study. As a result, the force data of two participants could not be analysed.

### Analysis of the contact mechanics

Detailed force analyses were performed on swipes for which the continuous motion on the surface was longer than one second and the exploration length was at least four centimetres. The continuous force measurements were segmented into single epochs corresponding to distinct phases within single swipes (Fig. 2a). To express the variations in tangential force during each swipe, the tangential force component was smoothed using a moving average filter with a 0.25 s time constant, and then differentiated. The onset of exploration (t_0_) was defined as the instant when the normal force reached a value greater than the maximum noise level of the force plate (40 mN). This time point corresponds to the moment when the force sensor measures a significant increase of normal force due to the finger pressing against the surface. The end of the exploration (t_4_) was defined as the instant when the first derivative of the tangential force reaches its minimum. This corresponds to the moment when the participant begins unloading the finger from the surface. Three successive segments were then defined (loading phase, partial slip and full slip). The loading phase segment started at the onset of exploration (t_0_). During this phase, the fingerpad remained in static contact with the surface, and an accelerating increase in tangential force was observed. The transition between the loading phase segment and the partial slip segment was defined as the instant when the rate of the tangential force reached a maximum (t_1_), as this sudden change in tangential force dynamics was thought to result from the occurrence of partial slip between the fingerpad and surface. The transition from partial slip to full slip was defined as the instant (t_2_) when the derivative of the tangential force became smaller than its standard deviation (estimated in the interval [t_1_, t_4_]). This was justified by the fact that once the finger starts sliding steadily against the surface, the tangential force remains relatively constant, provided that both the normal force and the dynamic coefficient of friction stay constant. Finally, the end of the full slip segment was defined as the time point (t_3_) when the derivative of the tangential force became more negative than its standard deviation estimated in the interval [t_1_, t_4_]. This last time point marked the end of the phase during which the finger was steadily sliding against the surface. Since the speed of exploration varied between trials, the force data measurements were converted to the spatial domain^20^. After this transformation, the length of the full slip segments were further restricted to two centimetres of sliding in order to match their duration with that of the transient phases. Since averaging the Spatial Fast Fourier Transform (SFFT) over all swipes for each participant required using identical epoch for each swipe, we computed the t_0_, t_1_ and t_2_ values for each participant over all its swipes, turned them into spatial coordinates and used their median values to implement the participant’s average SFFT of glass and PMMA.

### Statistics

The decision to use parametric or non-parametric statistical methods on given data sample was motivated by the D’Agostino and Pearson omnibus normality test, which we performed on all analysed samples using GraphPad Prism software.

## Additional Information

**How to cite this article**: Gueorguiev, D. *et al.* Touch uses frictional cues to discriminate flat materials. *Sci. Rep.* **6**, 25553; doi: 10.1038/srep25553 (2016).

## Supplementary Material

Supplementary Information

Supplementary Movie 1

## References

[b1] Jenmalm P. & Johansson R. S. Visual and Somatosensory Information About Object Shape Control Manipulative Fingertip Forces. J. Neurosci. 17, 4486–4499 (1997). 9151765 10.1523/JNEUROSCI.17-11-04486.1997 PMC6573538

[b2] Kappers A. M. L. Human perception of shape from touch. Philos. Trans. R. Soc. B Biol. Sci. 366, 3106–3114 (2011). 10.1098/rstb.2011.0171 PMC3172608 21969692

[b3] Westling G. & Johansson R. S. Factors influencing the force control during precision grip. Exp. brain Res. 53, 277–84 (1984). 6705863 10.1007/BF00238156

[b4] Bensmaïa S. & M. H. The Vibration of texture. Somatosens. Mot. Res. 20, 33–43 (2003). 12745443 10.1080/0899022031000083825 PMC2074877

[b5] Manfredi L. R. *et al.* Natural scenes in tactile texture. J. Neurophysiol. 111, 1792–802 (2014). 24523522 10.1152/jn.00680.2013

[b6] Goodwin a W., John K. T. & Marceglia A. H. Tactile discrimination of curvature by humans using only cutaneous information from the fingerpads. Exp. brain Res. 86, 663–72 (1991). 1761098 10.1007/BF00230540

[b7] Srinivasan M. A. & Lamotte R. H. Tactual discrimination of softness. J. Neurophysiol. 73, 88–101 (1995). 7714593 10.1152/jn.1995.73.1.88

[b8] Smith a M. & Scott S. H. Subjective scaling of smooth surface friction. J. Neurophysiol. 75, 1957–1962 (1996). 8734594 10.1152/jn.1996.75.5.1957

[b9] Ho H.-N. & Jones L. A. Contribution of thermal cues to material discrimination and localization. Percept. Psychophys. 68, 118–128 (2006). 16617836 10.3758/bf03193662

[b10] Tiest W. M. B. & Kappers A. M. L. Tactile perception of thermal diffusivity. Atten. Percept. Psychophys. 71, 481–489 (2009). 19304639 10.3758/APP.71.3.481

[b11] Moser K., Kriwet K., Naik A., Kalia Y. N. & Guy R. H. Passive skin penetration enhancement and its quantification *in vitro*. Eur. J. Pharm. Biopharm. 52, 103–112 (2001). 11522474 10.1016/s0939-6411(01)00166-7

[b12] Pasumarty S. M., Johnson S. A., Watson S. A. & Adams M. J. Friction of the human finger pad: Influence of moisture, occlusion and velocity. Tribol. Lett. 44, 117–137 (2011).

[b13] Hollins M. & Risner S. R. Evidence for the duplex theory of tactile texture perception. Percept. Psychophys. 62, 695–705 (2000). 10883578 10.3758/bf03206916

[b14] Andre T., Levesque V., Hayward V., Lefevre P. & Thonnard J.-L. Effect of skin hydration on the dynamics of fingertip gripping contact. J. R. Soc. Interface 8, 1574–1583 (2011). 21490002 10.1098/rsif.2011.0086 PMC3177614

[b15] Terekhov A. V. & Hayward V. Minimal adhesion surface area in tangentially loaded digital contacts. J. Biomech. 44, 2508–2510 (2011). 21774936 10.1016/j.jbiomech.2011.07.007

[b16] Delhaye B., Lefèvre P. & Thonnard J. Dynamics of fingertip contact during the onset of tangential slip. J. R. Soc. Interface 11, 20140698 (2014). 25253033 10.1098/rsif.2014.0698 PMC4191101

[b17] Gescheider G. A., Thorpe J. M., Goodarz J. & Bolanowski S. J. The effects of skin temperature on the detection and discrimination. 14, 181–188 (1997). 10.1080/08990229771042 9402648

[b18] Bolanowski S. J. & Verrillo R. T. Temperature and criterion effects in a somatosensory subsystem: a neurophysiological and psychophysical study. J. Neurophysiol. 48, 836–855 (1982). 7131055 10.1152/jn.1982.48.3.836

[b19] Klöcker A., Wiertlewski M., Théate V., Hayward V. & Thonnard J. L. Physical factors influencing pleasant touch during tactile exploration. PLoS One 8, 10.1371/journal.pone.0079085 (2013). PMC3828339 24244425

[b20] Wiertlewski M., Lozada J. & Hayward V. The spatial spectrum of tangential skin displacement can encode tactual texture. IEEE Trans. Robot. 27, 461–472 (2011).

[b21] Wiertlewski M., Hudin C. & Hayward V. On the 1/f noise and non-integer harmonic decay of the interaction of a finger sliding on flat and sinusoidal surfaces. *2011 IEEE World Haptics Conf. WHC 2011* 25–30, doi: 10.1109/WHC.2011.5945456 (2011).

[b22] Adams M. J. *et al.* Finger pad friction and its role in grip and touch. J. R. Soc. Interface 10, 20120467 (2013). 23256185 10.1098/rsif.2012.0467 PMC3565724

[b23] Derler S., Rossi R. & Rotaru G.-M. Understanding the variation of friction coefficients of human skin as a function of skin hydration and interfacial water films. Proc. Inst. Mech. Eng. Part J J. Eng. Tribol. 229, 285–293 (2014).

[b24] Dinç O. S., Ettles C. M., Calabrese S. J. & Scarton H. A. Some Parameters Affecting Tactile Friction. J. Tribol. 113, 512 (1991).

[b25] Adams M. J., Briscoe B. J. & Johnson S. A. Friction and lubrication of human skin. Tribol. Lett. 26, 239–253 (2007).

[b26] Jones L. A. & Lederman S. J. Human Hand Function. The Journal of Hand Surgery European Volume 32, 280, 10.1016/j.jhse.2007.04.015 (2006).

[b27] Bergmann Tiest W. M. & Kappers A. M. L. Analysis of haptic perception of materials by multidimensional scaling and physical measurements of roughness and compressibility. Acta Psychol. (Amst). 121, 1–20 (2006). 16055070 10.1016/j.actpsy.2005.04.005

[b28] Weber A. I. *et al.* Spatial and temporal codes mediate the tactile perception of natural textures. Proc. Natl. Acad. Sci. USA 110, 17107–12 (2013). 24082087 10.1073/pnas.1305509110 PMC3800989

[b29] Skedung L. *et al.* Feeling small: exploring the tactile perception limits. Sci. Rep. 3, 2617 (2013). 24030568 10.1038/srep02617 PMC3771396

[b30] Jones L. A. & Berris M. Material discrimination and thermal perception. *11th Symp. Haptic Interfaces Virtual Environ. Teleoperator Syst. 2003. HAPTICS 2003. Proceedings.* (2003). doi:10.1109/HAPTIC.2003.1191267.

[b31] Ho H. & Jones L. A. Material identification using real and simulated thermal cues. Conf. Proc. IEEE Eng. Med. Biol. Soc. 4, 2462–2465 (2004). 17270771 10.1109/IEMBS.2004.1403711

