# Supplementary material for: Touch uses frictional cues to discriminate flat materials
Source: Sci Rep. 2016 May 6;6:25553. doi: 10.1038/srep25553 (PMC4858763; doi:10.1038/srep25553)
Supplement: Supplementary Information [file srep25553-s1.pdf]

Touch uses frictional cues to discriminate flat materials

David Gueorguiev, Séréna Bochereau, André Mouraux, Vincent Hayward, and Jean-Louis Thonnard

SUPPLEMENTARY INFORMATION

Supplementary data 1: Profilometry of the materials' surface

Profilometric measurements were performed with a digital holographic microscope (Lyncée Tec, Switzerland) on four samples of each material to verify their flatness. The surfaces were extremely smooth (R_a glass = 8.1 10^{-6} mm; R_a PMMA = 6.2 10^{-6} mm) and did not exhibit salient topographic features beyond the nanometre scale.

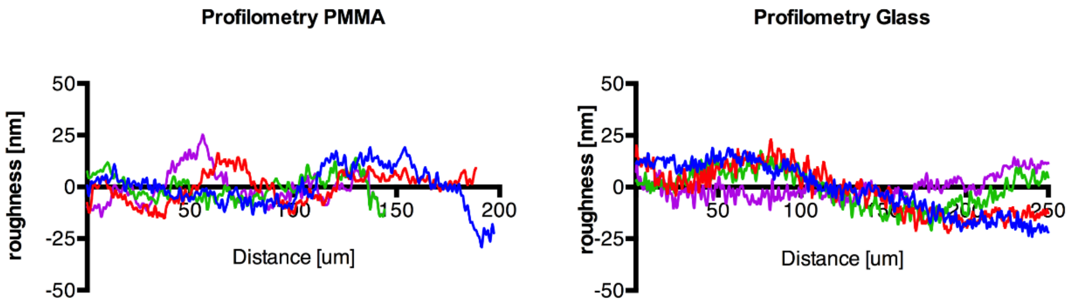

Supplementary Figure 1. Surface topography of the samples, related to materials and methods (subsection "Materials"). Height samples used during experiment 2 were randomly picked and assessed prior to the experiment: four samples of PMMA and four samples of glass.

Supplementary data 2: Homogeneity of performance across the experiments

The surfaces were shown to be quasi perfectly flat prior the experiment but a major concern during the two experiments was to avoid the influence of potential imperfections. In addition to the control procedure that was implemented during the experiments (See Materials and Methods: General procedure), the homogeneity of the performance across the different plates on which the samples were glued was also tested. Four distinct plates were used in each experiment to present all the possible orders since the plates with Glass – Glass – PMMA and PMMA – PMMA – Glass could be used for presenting different

orders of the samples. Thus, an imperfection on a plate could have impacted the responses for either one or two of the possible orders of presentation. In experiment 1, the performance was found to be different depending on the target material (Fig 1a.). We therefore investigated if this bias was due to an undetected imperfection or to a natural difference like, for example, a higher probability of a transient increase in the stickiness (stick and slip) for one of the materials. If the bias was due to an imperfection on a plate, it should have impacted the participants' answers for the scratched plate but repeated-measures ANOVA with Geisser-Greenhouse correction performed on all the possible orders where the target sample was PMMA ($F(1.262,13.88) = 0.806$, $p = 0.413$) and where the target sample was glass ($F(1.823,20.05) = 1.477$, $p = 0.25$) showed no inconsistency in the answers of participants (Supplementary Fig 2).

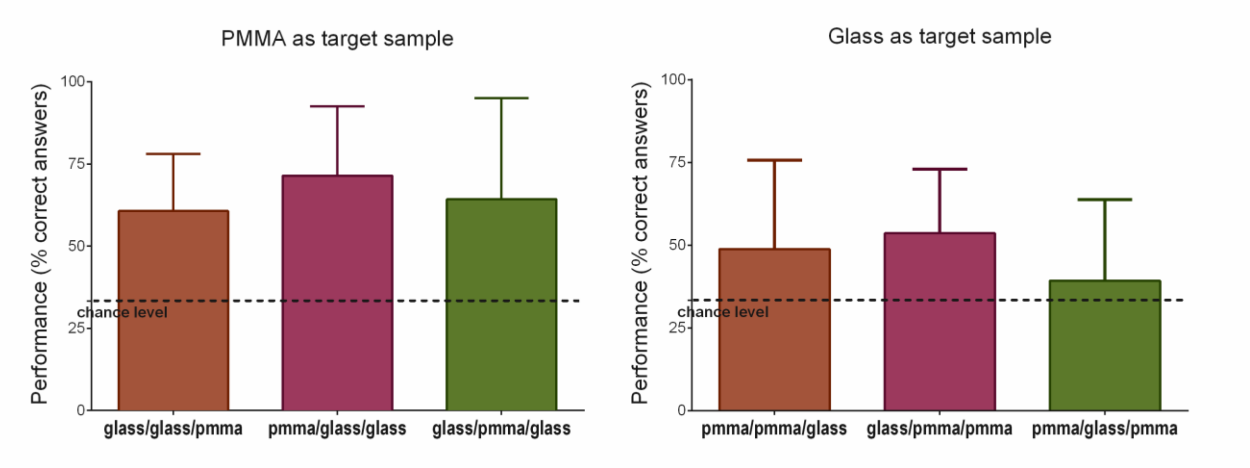

Supplementary Figure 2. Performance for each of the possible presentations of the stimuli in experiment 1. Performance (mean ± s.d.) was consistent across all possible orders where PMMA was the target sample (Left) and across all possible orders where glass was the target sample (Right).

In experiment 2, performance was similar for glass and PMMA but a non-parametric Friedman test showed a lower performance when the target sample was located in the middle ($\chi^2 = 11.87$, $p = 0.0026$). Thus, we tested if there was a discrepancy in the responses that would suggest the bias to be due to an imperfection rather than to the psychophysical criterion of the participants. Repeated-measures ANOVA with Geisser-Greenhouse correction was performed across all the plates where the target sample was at the distal or proximal location and showed no significant difference ($F(2.565,28.22) = 2.845$, $p = 0.063$). Wilcoxon matched-pairs signed rank test was performed between the two cases when the target sample

was located in the middle and showed consistent answers (12 participants: N=8, W=1, p = 0.99) (Supplementary Figure 3).

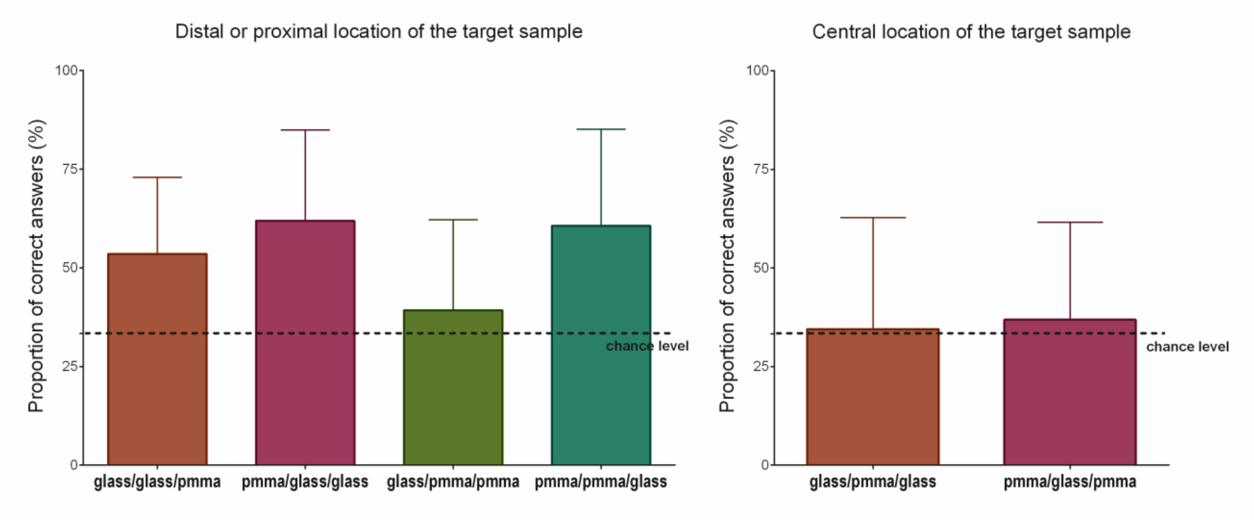

Supplementary Figure 3. Performance for each of the possible presentations of the stimuli in experiment 2. Performance (mean ± s.d) was consistent across all cases where the target sample was in a distal or proximal location (Left) and across all cases where it was centrally located (Right).

Supplementary data 3: behavioural parameters in experiment 2

Explorations were constrained during experiment 2 but the participants had a large amount of behavioural freedom particularly in terms of exploration speed, normal force applied, and possible changes of strategy, which could influence the differences in contact mechanics between the two materials (Supplementary Fig. 2). Despite the different strategies implemented by the participants, we did not find any influence of normal force (R = 0.03, p = 0.95; Pearson's correlation for n = 10) and mean duration of exploration (R = 0.04, p = 0.92; Pearson's correlation for n = 10). These results confirm the hypothesis that the capacity to discriminate relied on specific properties of the fingertip-surface interaction rather than on an optimal choice of exploring strategy.

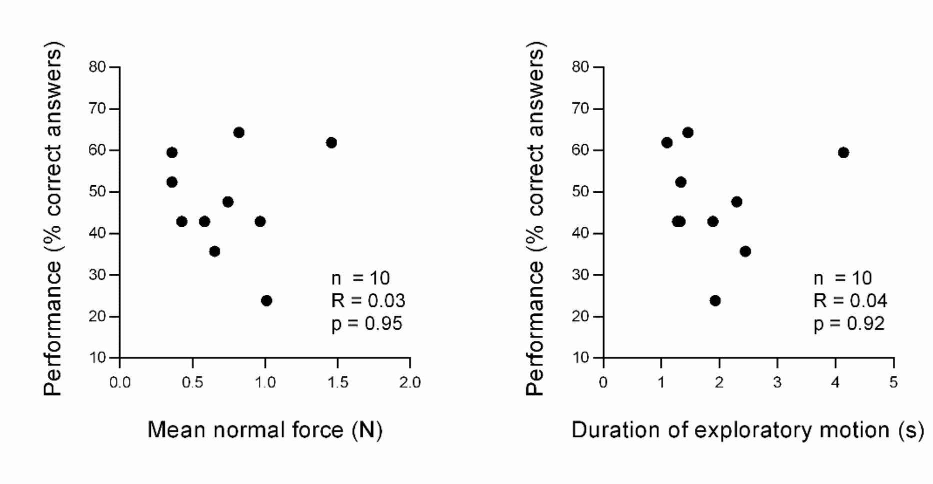

Supplementary Figure 4. Normal force and mean duration of the exploration as a function of performance, related to Figure 2. Left: The specific normal forces used by the each participants while performing the task did not correlate with their performance. Right: The mean duration of the exploratory motion during the task similarly did not affect the participant's performance.

Illustration of partial slip

Supplementary Movie 1. Partial Slip, related to the discussion about Contact Mechanics. During an active exploration of a glass surface, a high-speed camera situated under the glass filmed the contact area. The first second of the movie illustrates the mechanics of partial slip described in the 'Contact Mechanics' section of the discussion. We see the peripheral slip spreading non-linearly to the central contact region. One can also observe that the contact area slides uniformly during full slip, which occurred after approximately one second.
